# Overlapping Symptoms of COVID-19 and Giant Cell Arteritis: The Need for a Higher Degree of Suspicion for Diagnostic Differentiation

**DOI:** 10.7759/cureus.25660

**Published:** 2022-06-04

**Authors:** Binit Aryal, Nevil Kadakia, Aashish Baniya, Tutul Chowdhury, Samaj Adhikari, Nicole Gousy

**Affiliations:** 1 Internal Medicine, Interfaith Medical Center, Brooklyn, USA; 2 Neurology, Upendra Devkota Memorial National Institute of Neurological and Allied Sciences (UDM-NINAS), Kathmandu, NPL; 3 Medicine, American University of Antigua, New York, USA

**Keywords:** inflammatory rheu, large vessel vasculitis, large vessel vasculitis and gca, giant cell arteritis, covid 19

## Abstract

Giant cell arteritis (GCA) is a large cell vasculitis that can present with a plethora of symptoms affecting several different systems. Before the COVID-19 pandemic, diagnosis of GCA was straightforward since the list of differential diagnoses for this disease was relatively short. However, the development of a SARS-CoV-2 viral infection challenges this standard. COVID-19 is a viral illness that also can present with similar vascular symptoms as GCS and creates a substantial inflammatory reaction, similar to most vasculitis. We present a case of a patient who had developed GCA after recovering from a COVID-19 viral illness. This is a rare presentation of GCA in the setting of COVID-19, and recognition of the nuanced differences between the two diseases may significantly change a patient’s prognosis if not detected early.

## Introduction

Giant cell arteritis (GCA) is one of the more common idiopathic granulomatous, vasculitic emergencies in elderly people, exhibiting a heterogeneous clinical spectrum and carrying the risk of vision loss if untreated urgently [1]. GCA has a poorly understood pathogenesis along with a strong association with human leukocyte antigens (HLA) types I and II [1]. Histopathologically, GCA manifests as a granulomatous inflammatory infiltration of the vessel wall. On a similar note, an infection with the SARS-CoV-2 virus leading to a COVID-19 infection can involve multiple systems, especially the vascular system, due to the intense surge of inflammatory cytokines associated with the disease [1,2]. Despite the similar inflammatory damage induced by both GCA and the COVID-19 virus, very few cases have been reported regarding a potential association between GCA and COVID-19 [1-3]. There has been an increasingly elevated rate of COVID-19 cases diagnosed in concordance with other primary systemic vasculitis or cases of polymyalgia rheumatica; however, there are few cases of GCA being seen in the context of an ongoing COVID-19 infection [3,4]. During the period of the COVID-19 pandemic, clinicians expressed significant diagnostic challenges since a COVID-19 infection can present with incredibly similar symptoms to GCA, including headache, fever, and myalgia [1,3,4], thus making the clinical distinction between these highly different diseases increasingly difficult. Herein, we present a case of a patient who developed intractable left-sided headache strongly suggestive of GCA following COVID-19 infection with high erythrocyte sedimentation rate (ESR) but negative temporal biopsy. It is worth noting that the patient improved after a high dose of corticosteroid therapy and is currently under evaluation by a rheumatologist on an outpatient basis.

## Case presentation

Our patient is a 72-year-old female with a history of chronic obstructive pulmonary disease, asthma, hypertension, and hyperlipidemia, and a previous history of a gastrointestinal bleed, a cerebrovascular accident in 2014 resulting in residual left arm weakness and limited range of motion, and a history of a right lower extremity deep venous thrombosis (DVT) managed with inferior vena cava (IVC) filter in the past. She also has a history of bowel resection secondary to perforated diverticulitis, where she developed transient acute respiratory failure secondary to PCR-confirmed COVID infection. During investigation, her ESR was increased and her chest X-ray showed a band-like density in the left base consistent with atelectasis. She was managed conservatively without any COVID-specific treatment, steroids, or antibiotics, and was discharged home after symptom resolution after a week.

**Figure 1:**
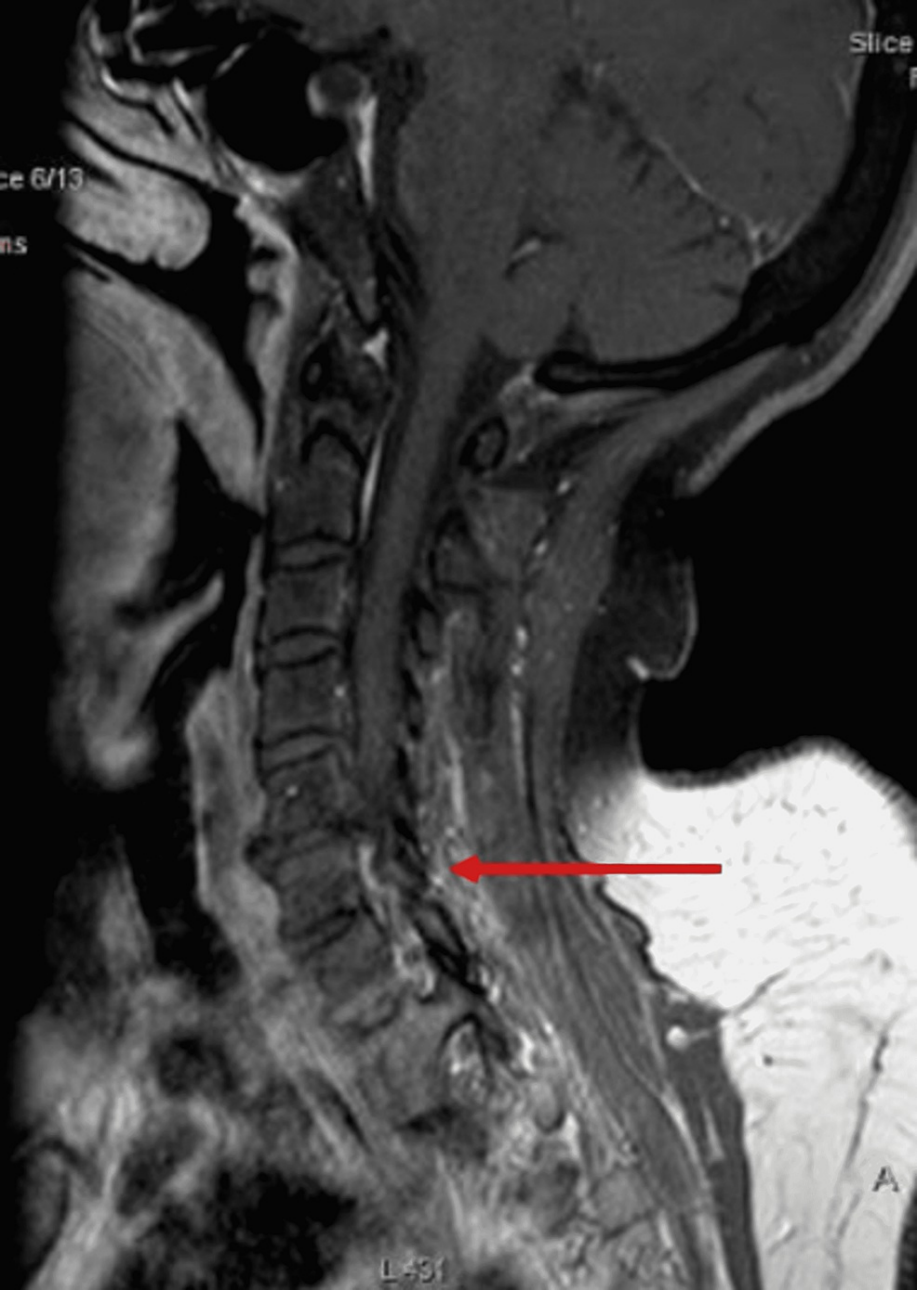
MRI of the cervical spine showing severe canal stenosis at C4-C5, C5-C6, and C6-C7, and multilevel severe neural foramina stenosis (red arrow).

After two months, the patient again presented to the emergency department for left-sided headache, blurry vision, intermittent vision loss in the left eye, dizziness, and giddiness, along with a non-specific abdominal pain. Upon investigation, her ESR was 66 mm/hour, C-reactive protein (CRP) was 6.5 mg/L, and thyroid-stimulating hormone (TSH) was 2.2 mIU/L. A head CT was performed and was negative for any acute hemorrhage or masses. Initial differentials were severe cervical canal stenosis, cerebrovascular accident, GCA, trigeminal neuralgia, and migraine and trapezius/occipital neuralgia. The pain management team was consulted, and after initial evaluation, they recommended commencing gabapentin. Further imaging of the head and neck and autoimmune workup was recommended by the neurology team. Her MRI of the brain showed no acute intracranial abnormality, mass, or hemorrhage, and MRI of the cervical spine showed degenerative changes superimposed upon congenital stenosis including severe canal stenosis at C4-C5, C5-C6, and C6-C7, and multilevel severe neural foramina stenosis (Figure 1).

Given the ophthalmic symptoms, a carotid Doppler was also performed, which showed bilateral internal carotid stenosis greater than 50% stenosis. Additionally, a transcranial Doppler was performed and subsequently was negative for stenosis. An autoimmune workup (including tests for double-stranded DNA IgG, Sjogren syndrome antibody A, Sjogren syndrome antibody B, Lyme’s disease antibody, rheumatoid factor, anti-cyclic citrullinated peptides, anti-neutrophil cytoplasmic antibodies, angiotensin-converting enzyme) were all negative or normal. Due to the gradual onset of clinical symptoms, which includes recurring left-sided headache, amaurosis fugax, and blurry vision, with risk factors including age, female gender, and hypertension, and significant lab findings of elevated ESR, a temporal artery biopsy for GCA was warranted and performed. However, biopsy results were negative. The patient was nonetheless treated as a case of biopsy-negative GCA and was administered IV steroids during hospitalization, after which, the ESR began to trend down. Upon discharge after a week, the patient was provided with oral prednisone with a tapering dose for two weeks. She followed up with the primary care physician, ophthalmology, and rheumatology services upon discharge. At the rheumatology Clinic, due to persistent symptoms, she was started on azathioprine 50 four times a day and Depo-Medrol 60 mg intramuscular while on prednisone 60 mg daily and was advised to repeat ESR in two weeks. However, she could not tolerate prednisone and azathioprine and hence both medications were discontinued and she was started on methotrexate 7.5 mg once/week and daily folate. After receiving steroid-sparing medication, her ESR decreased to 80 mm/hour from 60 mm/hour within a period of one month. As of her last visit, the patient now is asymptomatic and specifically denies any headaches or visual symptoms.

## Discussion

GCA and COVID-19 may initially present with similar symptoms. In a recent COVID-19 study, headache was reported in 2-34% of patients and fever in 83% of patients [4]. Recent systematic reviews and meta-analyses indicate that only around 66% of patients with GCA report headaches, while around 25% report fever [4]. Acute-phase reactants are elevated in both conditions [4,5]. Thrombocytosis was seen more with GCA, and lymphopenia with COVID-19. In a study conducted in China, the average duration of COVID-19 symptoms was one to two weeks. The average symptom duration in GCA is reported to be nine weeks but can be variable. On average, symptom duration is somewhat longer in non-headache presentations and shorter in those with isolated cranial symptoms [6]. Dry cough has been seen in a minority of patients with GCA [6]. Further evaluation of cough is necessary for patients presenting with new-onset GCA since this might be associated with the involvement of the aorta and its proximal branches. This is a potential risk factor for relapse or aortic aneurysm in GCA. Further studies need to be conducted on this for definite guidelines. Symptoms that coincide between COVID-19 and GCA need further evaluation. These symptoms include GCA-like symptoms, such as scalp tenderness, temporal artery tenderness, difficulty chewing, transient visual loss, weight loss, dysphagia, and trismus, and COVID-19 features such as dry cough, sore throat, dyspnea, confusion, anosmia or alteration in sense of taste lymphopenia, thrombocytopenia, elevation in lactate dehydrogenase, and elevation in creatine kinase [4,5]. Further studies are required to know if GCA patients with short symptom duration (days to weeks) differ from patients presenting with long symptom duration (months to years) [1,3]. Most new-onset headaches in the current clinical scenario are attributed to COVID-19 infection. However, given that GCA is seen in patients with COVID-19 infection, further evaluation for the diagnosis of GCA is necessary.

## Conclusions

The symptoms of GCA and COVID-19 may overlap, which include headache, fever, elevated CRP, and cough. However, Jaw claudication, visual loss, platelet count, and lymphocyte count may be more discriminatory. Therefore, clinicians should be aware of the possibility of diagnostic confusion. Further studies are required to know if GCA patients with short symptom duration (days to weeks) differ from patients presenting with long symptom duration (months to years). Most new-onset headaches in the current clinical scenario are attributed to COVID-19 infection. However, given that GCA is seen in patients with COVID-19 infection, further evaluation for the diagnosis of GCA is necessary. Few cases of GCA have been reported following COVID-19 infection. However, more studies are required to establish the relationship between COVID-19 infection and GCA.
